# Obtaining Hemocytes from the Hawaiian Bobtail Squid *Euprymna scolopes* and Observing their Adherence to Symbiotic and Non-Symbiotic Bacteria

**DOI:** 10.3791/1714

**Published:** 2010-02-11

**Authors:** Andrew J. Collins, Spencer V. Nyholm

**Affiliations:** Department of Molecular and Cell Biology, University of Connecticut, Storrs

## Abstract

Studies concerning the role of the immune system in mediating molecular signaling between beneficial bacteria and their hosts have, in recent years, made significant contributions to our understanding of the co-evolution of eukaryotes with their microbiota.  The symbiotic association between the Hawaiian bobtail squid, *Euprymna scolopes* and the bioluminescent bacterium *Vibrio fischeri* has been utilized as a model system for understanding the effects of beneficial bacteria on animal development.  Recent studies have shown that macrophage-like hemocytes, the sole cellular component of the squid host's innate immune system, likely play an important role in mediating the establishment and maintenance of this association.  This protocol will demonstrate how to obtain hemocytes from *E. scolopes* and then use these cells in bacterial binding assays. Adult squid are first anesthetized before hemolymph is collected by syringe from the main cephalic blood vessel.  The host hemocytes, contained in the extracted hemolymph, are adhered to chambered glass coverslips and then exposed to green fluorescent protein-labeled symbiotic *Vibrio fischeri* and non-symbiotic *Vibrio harveyi*.  The hemocytes are counterstained with a fluorescent dye (Cell Tracker Orange, Invitrogen) and then visualized using fluorescent microscopy.

**Figure:**
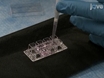


## Protocol

Prepare 500 mLs of 0.22 μm filter-sterilized artificial seawater (FSW; salinity 35 ppt). Filter artificial or natural seawater through a 0.22 μm micron filter to remove particles and bacteria.
Anesthetize one adult Hawaiian bobtail squid (*Euprymna scolopes*) by placing in a 2% solution of ethanol in FSW. Place the animal in anesthetic for approximately 10 minutes. The squid will cease swimming and will not actively respond to touch. Continued respiration, indicated by movement of the mantle, and chromatophore activity should still be observed.
Place squid with ventral side facing up on a standard wax dissection tray. Submerge the animal with FSW containing 2% ethanol.
Using one standard 200 μl pipette tips, pull back the funnel and mantle to expose the main cephalic blood vessel located between the two eyes.
Using a sterile 1 mL syringe with 26.5-gauge needle, puncture the cephalic blood vessel and withdraw between 50-100 μl of hemolymph. Place the hemolymph in a sterile 1.5 mL tube on ice.Note: If an animal will serve as a donor multiple times, only withdraw 10-20 μl of hemolymph at any given time. Return the animal to a normal seawater tank. The animal will revive within 30 min.
Freshly collected hemocytes are washed and re-suspended in 500 μl of Squid Ringer s solution (S-Ringers; 530 mM NaCl, 10 mM KCl, 25 mM MgCl_2_, 10 mM CaCl_2_ and 10 mM HEPES buffer, pH 7.5).
Hemocyte concentrations are determined by hemocytometer, and approximately 2,000 cells are added to chambered glass cover slips, and allowed to adhere to the glass for 10 min at room temperature. At this density, the hemocytes form a uniform monolayer on the glass slide surface.
To observe bacterial binding to host hemocytes, hemocytes are exposed to a fluorescently labeled bacterial strain such as *Vibrio fischeri* ES114 and/or *Vibrio harveyi *B392, each containing a green fluorescent reporter. *V. fischeri* ES114 and *V. harveyi* B392 are grown to mid-log phase in a sea water tryptone media (SWT) at 28°C in an orbital shaker. The optical density at 600nm is measured spectrophotometrically to determine cell density. The bacteria are pelleted by centrifugation (5,000 rpm for 5 min), the supernatant is discarded, and the pellet is re-suspended in S-Ringers.
100,000 bacterial cells are added to each chamber well so that there are 50 bacteria per hemocyte on average. The hemocyte/bacteria mixtures are incubated in S-Ringer s solution at 25°C for 1 h, a time determined to yield the maximum level of binding.
The cytoplasm of the hemocytes are then fluorescently stained with 0.005% CellTracker Orange (Invitrogen) and then washed in S-Ringers to visualize the cells.
Stained hemocytes with associated bacteria are viewed by fluorescence using either a Zeiss Discovery V20 fluorescent stereoscope or a Leica SP2 spectral laser confocal microscope, and enumerated over the entire surface of the animal cell.

### Representative Results

Because cephalopod hemolymph contains extracellular hemocyanin and not hemoglobin, upon oxygenation, the hemolymph will turn dark blue. An average of ~5000 hemocytes per μl of hemolymph will be obtained using this method. After adherence to the chambered cover slips and fluorescent staining, the hemocytes should appear brightly fluorescently red and amoeboid in shape. For bacterial adhesion, *V. fischeri* will adhere poorly to the hemocytes (1-2 bacterial cells per blood cell) while *V. harveyi* will adhere strongly (10-15 bacterial cells per hemocyte).


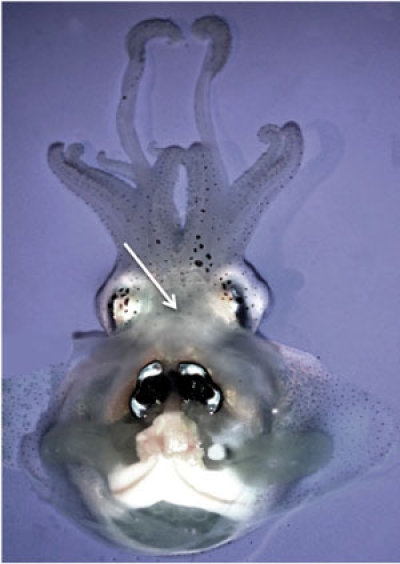
 **Figure 1.** Adult Hawaiian bobtail squid *Euprymna scolopes* showing position of cephalic blood vessel.


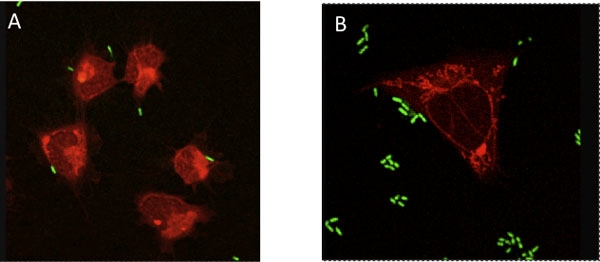
 **Figure 2.** Results of hemocyte exposure to *Vibrio fischeri* (A) and *Vibrio harveyi* (B). Red, Cell Tracker Orange; Green, GFP-labeled bacteria.

## Discussion

Studies concerning the role of the immune system in mediating molecular signaling between beneficial bacteria and their hosts have, in recent years, made significant contributions to our understanding of the co-evolution of eukaryotes with their microbiota.  The squid/vibrio system has proven itself as a tractable model system to answer fundamental questions in this field^2,3,5,6,8^. The light-organ of the squid *Euprymna scolopes* permits colonization exclusively by the luminous bacterium *Vibrio fischeri*.  Because the tissues that house the bacteria remain in contact with seawater, the squid must not only foster the specific symbiosis but also continue to exclude other bacteria. Continued studies have revealed that macrophage-like hemocytes likely play an important role in the establishment and maintenance of this association^1,4,7^. Because the squid host lacks adaptive immunity, the amazing specificity found in this association must be whole or partially mediated through the innate immune system. A recent investigation of these blood cells revealed that hemocytes isolated from *E. scolopes* recognize and phagocytose *V. fischeri* and non-symbiotic bacteria differentially and that colonization likely leads to a type of "immune tolerance" of the symbionts^4^. This protocol will demonstrate how to successfully obtain these blood cells from adult squid and test their ability to bind bacteria.
